# Teaming up for long-term care: Recognizing all long-term care staff contribute to quality care

**DOI:** 10.1177/08404704221115811

**Published:** 2022-09-15

**Authors:** Pat Armstrong, Hugh Armstrong, Ivy L. Bourgeault

**Affiliations:** 1 7991 York University, Toronto, Ontario, Canada.; 2 6339 Carleton University, Ottawa, Ontario, Canada.; 3 151181 University of Ottawa, Ottawa, Ontario, Canada.

## Abstract

When looking to promising international approaches to improve quality care in long-term care, it is necessary to avoid cherry-picking specific dimensions ignoring the integrated nature of what makes these approaches promising in the first place. In looking at promising Scandinavian or Green House models, attention is often paid to the size of facility. This often overlooks the importance of higher level of staffing, mix, and compensation of direct care staff and the integration of dietary, laundry, and housekeeping staff to care teams. Other overlooked considerations include recognition of family and friends and policies supporting care continuity.

## Introduction

In the wake of the disastrous impact COVID-19 had on long-term care in Canada, there has been a search for ways to transform and enhance quality care (Table 1) in long-term care, including by those who hold leadership positions in these facilities. Increasingly that search has looked to what is often called the Scandinavian or U.S. *Green House* models,^1^ with particular attention to the smaller size of many homes compared to those in Canada. Although size of facility matters, our various research projects in Canada and abroad^2^ suggest other factors that are at least as important not only in saving lives but also in making life worth living. These are staffing levels, mix, and compensation. A long-term care home is only a building without staff to provide care. In contrast to Canada, both the Scandinavian and Green House models start with much higher staffing levels, with those in Sweden being two to three times higher.^3^ Drawing upon data gathered over a decade pre-pandemic involving in-depth interviews and site visits across dozens of facilities in Canada^4^ and internationally^5^ (Table 2), we highlight the importance of staffing levels, mix, and compensation for consideration by leaders in long-term care.

**Table 1.** Application of multi-dimensional conceptualizations of quality care^6,7^ as applied to long-term care.

Safe	Care provided ensures that residents and workers are physically, psychologically, and culturally safe
Effective	Care provided is based on a range of types of evidence
Patient- and family-centred	Care provided is responsive to residents’ and families’ preferences, needs, and values
Accessible and timely	Care is provided at the right time and right setting as needed
Efficient	Care provided should be mindful of the use of resources, such as supplies, equipment, and time
Equitable	Care should be provided according to needs
Integrated	Care provided should be organized and connected to other sectors from primary care to acute care

**Table 2.** Methodological details of two studies on which this commentary is based.

Study	Methodological details
They deserve better study	A survey of 948 workers (87.6% response rate) from three Canadian provinces and 1,625 workers (72% response rate) across four Nordic countries (Denmark, Finland, Norway, and Sweden).^2^
Seniors Adding Life to Years (SALTY) study	Over 1,000 hours of observations and 252 formal interviews with managers, staff, residents, family members and volunteers in eight non-profit or public homes two in each of four provinces—Nova Scotia, Ontario, Alberta, and British Columbia.^4^

## Level, mix, and compensation of direct care staff

Concerns around staffing levels, mix, and compensation have received considerable attention in various reports^8^ as well as in practice in Canada. Indeed, there are 196 references to “staffing” in the *Final Report* of Ontario’s Long-Term Care COVID-19 Commission.^9^ But the Commission is far from alone in highlighting the critical role staffing levels played in the pandemic’s terrible impact on long-term care. Canadian governments are being pushed to hire more nurses and care aides, of which the latter group provides most of the direct personal care. The Ontario government is promising an average of 4 hours of direct nursing care per resident per day. Similarly, the Quebec government promised 10,000 new jobs for what they call “Patient Care Attendants.”

Intricately connected with low staffing levels is the low pay for those who provide most of the direct nursing care; this issue was also recognized and addressed in many jurisdictions, at least in the short term. The federal government provided funds to provinces and territories to increase wages of “essential” workers in long-term care during the pandemic.^10^ Nova Scotia is increasing salaries for what they call “Continuing Care Assistants” by 23%.^11^ Quebec promised “a starting wage of $26 an hour, $49,000 annually, to the 10,000 people who complete the three-month program.”^12^ Despite these remarkable increases, these wages remain lower than in Scandinavia.

The mix of workers in long-term care, or skill mix, has also been raised as an issue that must be addressed, with calls for more registered nursing staff—and for additional professionals, such as physical and occupational therapists, social workers, and physicians, especially for the provision of palliative care. In Ontario, RNs account for 8% of the long-term care workforce, compared to 32% in Norway. Those with at least a year of formal education constitute 17% of workers in Ontario but 44% of healthcare workers in Norway and 59% of assistant nurses in Sweden.

Unquestionably, greater numbers of direct care staff, as well as a higher mix of professional-level staff are required, and unquestionably they need better pay and working conditions, as all the investigations into long-term care make clear.

## Who’s missing from these considerations?

Missing from much of this discussion about staffing models, policies and practices, are the other essential workers in long-term care teams that are central to care in Scandinavia and the Green House Model. The Ontario Long-Term Care Commission acknowledges that:

The pandemic brought the often under appreciated work and dedication of cooks, dietary aides, and housekeeping staff to the forefront. A COVID-positive case or absence of staff in these areas could quickly bring a home to the brink of collapse.

The reports from members of the Canadian military who were called upon to enter long-term care homes in crisis during the pandemic made it clear how the absence of appropriate food and assistance with eating, of clean laundry and assistance with dressing, and lack of housekeeping put everyone at risk. Currently, there is little policy and only limited managerial practices addressing those who do this work, although there is much to learn from other leading models.

Food is not only essential to ensuring residents absorb sufficient nutrients, as we have seen in many long-term care homes, meals are often the main event of the day, including for those who need assistance with eating. Meals can offer the opportunity to socialize, to see, chat with, and enjoy other residents. The smell of cooking can tempt older people with small appetites to eat. Food can bring pleasure in texture and taste if sufficient attention is paid to preparation and presentation.

Clean clothes are not only essential to prevent infection, rashes, and discomfort, clothes also provide a sense of identity and dignity of those who live in long-term care. Fresh linens can bring pleasure, and dignity too, while dirty, smelly ones bring the reverse, as well as the obvious risk of infection. Families and residents often tell us how important it is to be dressed each day and dressed in the clothes they brought with them to the home.

Housekeeping is also about more than preventing infection and other risks like falls. Housekeeping helps avoid smells and provide a more pleasant atmosphere, one where people want to live, work, and visit. Clean, pleasant environments are also about dignity and respect.

In the many care homes we have studied, dietary, food, laundry, and housekeeping workers do much more than the obvious tasks that come with their jobs. They also provide what is usually called *social care*, a form of care which needs to be recognized as essential. They chat with residents and their families. They listen to them. They help them get assistance when they need it, finding their glasses or finding a direct care staff member. They adapt their work to the individual residents and families they have come to know. In short, they are essential participants in the care team. Their work can help make a facility not only a safe place, but also a home, preventing unhealthy isolation.

## Care team recognition, contracting out, and continuity

In Scandinavia, those who do this work are considered integral members of the care team. As is the case with the Green House model, they are employees of the home. Indeed, there is frequently a less rigid division of labour among the direct care nursing staff and those who do the dietary, laundry, and service work.^13^ We witnessed regularly scheduled team meetings that involved all the staff, meetings that allowed everyone to contribute to discussions of how to respond to individual residents’ needs.

By way of contrast, in Canada, the food, laundry, and housekeeping services are often contracted out, and in some cases out to different companies. Having different employers and lines of accountability makes it difficult to be or feel part of a team. Moreover, the often for-profit nature of the companies contracted to provide the service are less likely to be interested supporting teamwork across jobs or the kind of social care that makes the job more satisfying for workers, residents, and families. Having the same employers for those who work in a home also supports the approach to care as a relationship that is more emphasized in the Green House model than in most Canadian long-term care.

Part and parcel with the increase in contracting out services are other strategies that promote more part-time, casual, and precarious employment.^14^ These practices disrupt continuity in care and care relationships as well as the teams that are central to both. To limit the spread of COVID-19 during the pandemic, several jurisdictions introduced polices that temporarily prevented part-time workers moving from one long-term care home to another in a search of full-time compensation. Few jurisdictions have addressed the conditions that motivate this movement in the first place; practices which have been implemented more so in Canada than in Scandinavian countries.

Alongside teamwork in the various models comes significant autonomy for staff. Our survey of direct care staff in Scandinavian and Canadian long-term care homes found that the Canadians were more than six times as likely as their Scandinavian counterparts to say they faced violence on a more or less daily basis.^2^ The resident populations were very similar but in the Nordic countries the staff had more time and more autonomy in responding to individual resident needs, which helps to avoid triggers for violent responses to rushed care.

## Other overlooked care team members

The pandemic not only exposed the importance of those paid to do the food, housekeeping and laundry work, it also exposed how much unpaid work is done in long-term care in Canada and how such care - as well as those who do it - is essential to the team. The work done by family, friends, and volunteers has become more obvious paradoxically at the same time as it was limited. Initially, they were banned from entering homes based on the assumption that they would spread infections, but it quickly became clear that their absence translated into a remarkable reduction in essential care.

Family and friends are encouraged (in some cases, expected) to offer social support and connections for residents in Canada, Norway, and Sweden. They are sources of knowledge about the resident’s past health experiences as well as their individual interests and tastes. In our Canadian research, we saw families and others brush teeth, comb, and even wash hair, assist with eating, dressing, and walking, and clean rooms, bums, and beds. They alert staff about urgent needs of their relative and of other residents, arrange activities, and entertain, to name only a few of their contributions.

The difference is not simply reflected in attitudes or family responsibility. It is also about the major differences in staffing and funding. In Sweden and Norway, paid staff are expected to provide all the other essential care, including social and emotional support. The resources are provided to enable them to do so, resources that are at least as important as the size of long-term care homes and the units within them.

The Canadian families, friends and volunteers we interviewed want to be involved in the nursing homes and to have the right to participate, and to be part of the care team. But they also want the care to be there when they are not, and they want the most intimate care to be provided by staff trained to do this skilled care work.

## Other overlooked considerations

It is also important to note that the Scandinavian and Green House models include private rooms and bathrooms for almost all the residents. In Canada, by contrast, residents often share a room, sometimes with as many as three others although this varies by jurisdiction. For example, “residents in Ontario were more likely to reside in shared rooms (63% of residents) than those in British Columbia (24%)”.^15^ Research in Ontario found that crowding in rooms made residents more susceptible to COVID-19, and that crowding was more common in for-profit long-term care homes.^16^

Other often overlooked dimensions are the significant differences in the for-profit ownership of long-term care homes. A high proportion of homes in Canada, and especially Ontario and BC, are owned by for-profit corporations and death from COVID-19 was much higher in them compared to non-profit or municipal homes.^17-19^ Sweden has relatively few for-profit homes and Norway even fewer, in part because they have actively worked to limit their extent in the wake of evidence on lower quality care.^20^

Indeed, the size of the rooms and the privacy they provide, along with for-profit status may be more important than the size of the long-term care home—as distinct from the size of the units within it—in preventing COVID-19. Research during the pandemic showed that Green House nursing homes in the US are less likely to be for-profit, to pay more to certified nursing assistants, and to have fewer residents who are confined to bed or are catheterized.^21^

In our Canadian interviews, it was not so much size as location that was most important to residents, families, and staff. They wanted the home to be near people and to have good public transport. We often heard residents wanted to be near activities and their former homes. We did not hear any support for the 32-bedroom units along a hospital-like corridor common in Canada. Those who called for smaller homes seldom specified how small. Like the Eden Alternative model, Scandinavian long-term care homes often have small households within much larger structures that allow residents to live closer to home in urban settings.

## Conclusion

In sum, building smaller homes is not the only lesson to learn from Scandinavian care homes or from the Green House model in the United States. Higher staffing levels and ratios to residents, with everyone working as an employee of the home and recognized as part of the team, are critical to promoting high quality care in long-term care. Full-time employment, improved working conditions and greater staff autonomy, enabling them to use their knowledge of residents to better respond to their unique needs are also necessary. Families, friends and volunteers need to be recognized and supported as part of the team, but not in a way that is dependent on their contributions to fill gaps in care. Leaders in long-term care need support and encouragement to recognize the importance of all staff in delivering high-quality long-term care, applying lessons learned from the pandemic and international comparisons.
